# Epoxy Adhesive Materials as Protective Coatings: Strength Property Analysis Using Machine Learning Algorithms

**DOI:** 10.3390/ma18122803

**Published:** 2025-06-14

**Authors:** Izabela Miturska-Barańska, Katarzyna Antosz

**Affiliations:** 1 Department of Production Computerisation and Robotisation, Faculty of Mechanical Engineering, Lublin University of Technology, Nadbystrzycka 36, 20-618 Lublin, Poland; i.miturska@pollub.pl; 2 Faculty of Mechanical Engineering and Aeronautics, Rzeszow University of Technology, 35-959 Rzeszow, Poland

**Keywords:** material characterization, SEM, machine learning algorithms, SVM, NN algorithm

## Abstract

This study analyzed the mechanical properties of epoxy adhesive materials used as functional coatings, focusing on how physical modifications impact their microstructure and strength. Compositions based on Epidian 5, 53 and 57 resins were cured using TFF, Z-1, or PAC curing agents and modified with various fillers: mineral (CaCO_3_ calcium carbonate), active (activated carbon filler, CWZ-22), and nanostructured (montmorillonite, ZR-2) fillers. The best results were achieved with calcium carbonate (10–20 wt%) in Epidian 5 or 53 resins cured with TFF or Z-1, yielding tensile strength up to 64 MPa, compressive strength up to 145 MPa, and bending strength up to 123 MPa. Activated carbon and nanofillers showed moderate improvements, particularly in more flexible matrices. To support property prediction, machine learning algorithms were applied and successfully modeled the mechanical behavior based on composition data. The most accurate models reached R^2^ values of 0.93–0.95 for compression and bending strength. While the models for compression and bending strength demonstrated high accuracy, the tensile strength model yielded lower predictive performance, indicating that further refinement and expanded input features are necessary. Shapley analysis further identified curing agents and fillers as key predictive features. This integrated experimental and data-driven approach offers an effective framework for optimizing epoxy-based coatings in industrial applications.

## 1. Introduction

Modern engineering structures increasingly demand materials that can simultaneously fulfill structural and protective roles. In mechanically and chemically aggressive environments, coatings serve as a vital barrier to moisture, corrosion, UV radiation, and mechanical damage. Depending on their chemical composition and technical function, coatings can perform barrier, adhesive, electro-insulating, or self-healing functions [1,2].

Among these, epoxy adhesive systems are particularly versatile. Traditionally used as structural adhesives, they are also employed as protective coatings for substrates such as steel, concrete, and composites. These two-component polymeric systems form cross-linked networks with excellent adhesion, chemical resistance, and thermal stability after curing. Their properties can be tailored through appropriate selection of curing agents and fillers [3,4].

In various engineering applications—including construction, marine, automotive, and energy—epoxy coatings are valued for their adaptability and durability. They also serve as the foundation for smart coatings and hybrid materials [5].

One established method for enhancing epoxy performance is physical modification using mineral, active, or nanostructured fillers. These improve characteristics such as modulus, toughness, and barrier performance. Fillers like calcium carbonate, activated carbon, and montmorillonite have demonstrated measurable influence on microstructure and mechanical behavior [4,5,6]. However, the effectiveness of such modification depends on filler dispersion and interaction with both the resin and curing agent.

Thus, the use of various types of fillers—both macro-, micro-, and nano-structural, which in specific proportions can significantly improve the mechanical, thermal, and tribological properties of composite systems—is a key strategy for modifying epoxy resins. Studies by Karthikeyan and Naveen [4] have shown that mineral additives such as CaCO_3_ can improve elastic modulus and compressive strength, while active carbon fillers (e.g., CWZ-22) can have beneficial effects on vibration damping and thermal conductivity. On the other hand, the use of nanofillers—such as montmorillonite of the ZR-2 NanoBent type—can achieve better barrier properties and significant reinforcement at a low mass proportion (1–5%) [7].

Scanning electron microscopy (SEM) is often used to assess the resin–filler interface, as poor dispersion can result in porosity or reduced mechanical performance [8]. At the same time, traditional mechanical testing of new formulations is often time-consuming and expensive.

Another challenge remains the rapid and reliable evaluation of the mechanical properties of new compositions, which traditionally requires time-consuming and expensive experimental testing. To address this, machine learning (ML) algorithms are increasingly employed to predict and optimize material properties. These include linear models, random forests, and neural networks, which can identify key formulation parameters and forecast strength, durability, or failure [9,10,11]. For example, Yan et al. [9] used the ML model to predict the tribological properties of epoxy coatings modified with nanoparticles, achieving high prediction accuracy. Other researchers [10] developed regression models to evaluate the strength of adhesive bonds depending on resin type, filler type, and curing method, indicating the potential of AI in the design of new materials.

Despite the numerous studies on epoxy resins and the widespread interest in the topic of modification of polymeric materials, there is still a lack of research that comprehensively combines experimental strength analysis, microstructural characterization, and predictive modeling of mechanical properties for epoxy protective coatings. This is particularly true for resins in the Epidian group, which are widely used in industry, but insufficiently described in the context of ML-supported physicochemical modifications.

Due to the challenges in directly testing thin coatings, model samples of standardized geometry were used, allowing consistent evaluation of compositional effects. This type of approach allows unambiguous evaluation of the influence of epoxy resin, curing agent, and filler type on mechanical properties, eliminating uncontrolled factors related to the substrate, uneven film thickness, or curing conditions in thin films. Although the samples used do not fully replicate the in-situ operating conditions of the coatings, they provide a reliable model to evaluate the material properties as such. A similar approach is commonly used in the literature on adhesion and coating testing of epoxy materials [12,13,14].

In response to the indicated research gap, the purpose of this study was to conduct a multivariate analysis of epoxy adhesive materials used as protective coatings, taking into account the following:

- the effect of resin type, curing agent type, and filler concentration and type on mechanical properties;
- evaluation of microstructure using scanning electron microscopy (SEM);
- construction and validation of regression models that enable prediction of strength parameters based on material composition.

The overarching goal of the paper is therefore to integrate experimental approaches with machine learning algorithms in the context of functional adhesive coatings based on Epidian epoxy resins.

## 2. Numerical Methods and Data Algorithms in Epoxy Materials Engineering

Advances in epoxy materials today are no longer solely the result of experimental research. Increasingly, the development of adhesion and coating technologies is based on the integration of classical laboratory approaches with computational methods and data analysis. In particular, numerical methods and machine learning algorithms are finding application in adhesion materials engineering as tools for predicting, optimizing, and deeply understanding the effects of chemical composition and microstructure on the mechanical and performance properties of epoxy composites.

The development of this field is made possible by the increasing availability of experimental data, the digitization of research, and the growth of computing power. Of key importance here are regression algorithms, classification methods, and data mining techniques that allow identification of non-obvious relationships between variables such as resin type, curing agent type, filler concentration, or composite preparation method. The most commonly used algorithms are linear and multivariate regression, Ridge and LASSO regression, decision trees, and ensemble models such as random forests (random forest). More advanced applications also use deep neural networks (DNNs) and convolutional neural networks (CNNs), especially for microscopic image analysis (e.g., SEM) [15,16,17].

In the engineering of epoxy adhesion materials, data-driven predictive models make it possible, among other things, to predict tensile strength, flexural and compressive strength, and to assess degradability under service conditions. In a study by Yan et al. [9], the use of a machine learning model made it possible to predict the tribological properties of epoxy coatings with high accuracy based on material composition and curing conditions. Another work (e.g., Jang et al. [10]) has demonstrated the effectiveness of regression in evaluating the effect of fillers on the mechanical parameters of structural adhesives.

**Figure 1:**
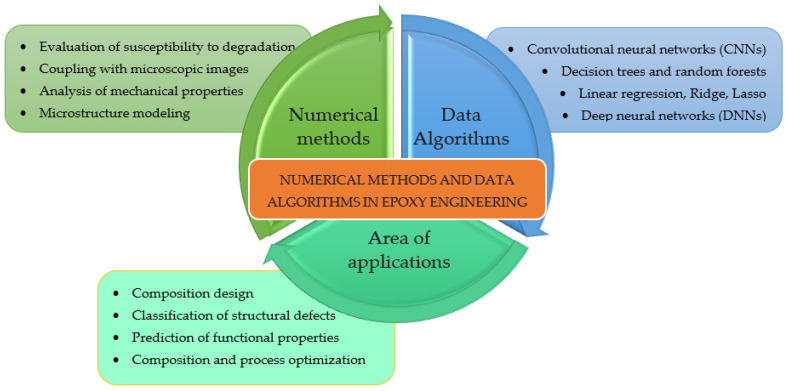
Numerical methods and data algorithms in epoxy engineering.

Particularly important in the case of epoxy resins is to take into account the microstructural variability resulting from the type of fillers (mineral, active, nano) and their dispersion in the polymer matrix. The use of numerical methods makes it possible to quantitatively analyze this variability and account for it in model predictions. Data-based models can also be coupled with microscopic observations, allowing not only the classification of structural defects but also their automatic assignment to technological parameters [6,18]. Figure 1 shows a modern approach to the analysis and design of epoxy materials using computational tools and artificial intelligence methods. The diagram illustrates how extensively numerical methods and data algorithms support modern epoxy engineering—from property analysis and prediction to intelligent design of materials with preplanned parameters. It also points to the growing integration of experimental approaches with digital analytical tools as part of the so-called digital transformation of materials research.

In this study, machine learning models were used to support the analysis of the influence of input variables on the mechanical properties of epoxy composites. Such a choice, despite its simplicity, is fully justified—especially for studies where sample size is limited and the goal is to understand the influence of specific formulation parameters. These models, properly calibrated and validated, make it possible to predict strength properties based on material composition, supporting both experimental analysis and practical application of materials in surface engineering.

The use of numerical methods and data algorithms in the engineering of epoxy adhesive materials not only speeds up the testing process but also enables the development of functional coatings with engineered properties tailored to the requirements of specific working environments. This represents a clear step toward so-called smart materials design and the digital transformation of materials research.

## 3. Materials and Methods

### 3.1. Materials and Their Properties

The study used epoxy composite compositions based on three different Epidian resins: Epidian 5, Epidian 53, and Epidian 57 (CIECH S.A., Sarzyna, Poland), which differ in the degree of chemical modification, viscosity, and elasticity after curing. Epidian 5 epoxy resin is a classic, low-viscosity bisphenol resin with a wide range of engineering applications. Epidian 53 epoxy resin, which is an ester-modified resin, is characterized by higher hardness and heat rejection. Epidian 57 epoxy resin, on the other hand, contains additives that improve the plasticity of the composition, making it more flexible after crosslinking.

Three different curing agents were used: TFF Mannich’s principal, Z-1 aminoamide and the PAC traditional polyamide curing agent (CIECH S.A., Sarzyna, Poland). Each of them is characterized by a different crosslinking mechanism and reaction time, which translates into different final properties of the material. Crosslinking reactions were carried out under room conditions, according to the manufacturers’ recommended ratios, keeping the appropriate gelation and curing times.

The properties of the used materials are summarized in Table 1 and Table 2.

The content in the epoxy compositions of the curing agents used was selected on the basis of the recommended stoichiometric ratios (Table 3), according to the type of epoxy resin.

In order to evaluate the effect of physical modification of the epoxy compositions, the compositions were enriched with three types of fillers: nanofiller (montmorillonite type NanoBent ZR2) (Zakłady Górniczo-Metalowe “Zębiec” S.A., Zębiec, Poland), mineral (calcium carbonate—CaCO_3_) (Zakłady Przemysłu Wapienniczego Trzuskawica S.A., Siatkówka, Poland) and activated (activated carbon CWZ-22) (PPH STANLAB SP. Z O.O., Lublin, Poland). Fillers were introduced into the resins at different ratios: 5%, 10% and 20% by weight for CaCO_3_ and CWZ-22, and 1%, 3% and 5% by weight for montmorillonite. Their selection was based on the previous literature reports indicating their cor- cussive effects on the mechanical and barrier properties of the epoxies.

The properties of the fillers used in the study are summarized in Table 4, Table 5 and Table 6. Table 7, Table 8 and Table 9 present the compositions of the compositions tested in the study.

So, in this study, compositions labeled as in Table 7 were prepared and tested.

### 3.2. Preparing Samples for Testing

**Figure 2:**
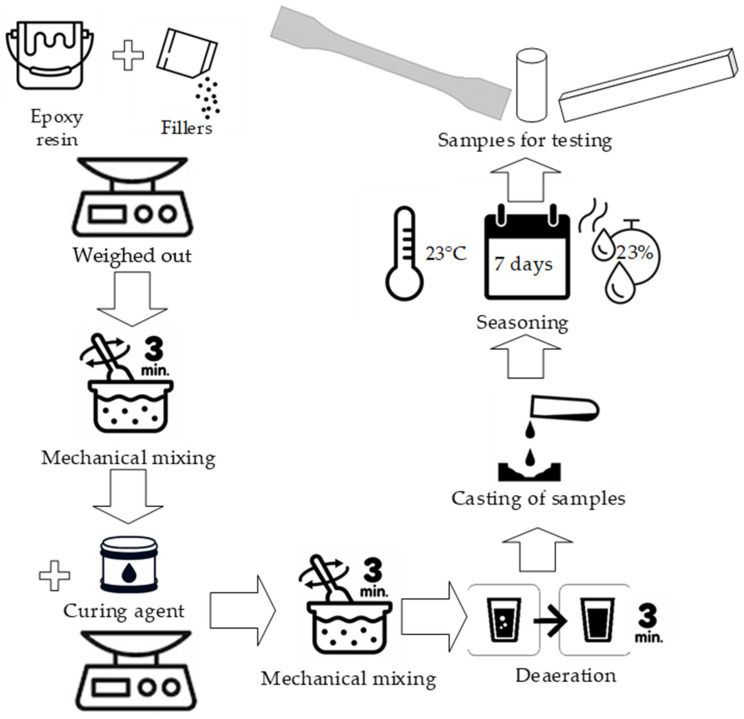
Flowchart of the test sample preparation process.

Preparation of the compositions was carried out using a mechanical mixer (Güde, Wolpertshausen, Germany) with a dispersing disc mixer. The flow of the process of preparation of epoxy compositions was carried out according to the following methodology (Figure 2):

- Weighing the specified amount of resin;
- Adding a filler (for modified compositions) to the epoxy resin in the appropriate amount;
- Mechanical stirring of the composition components for a period of 3 min at a speed of 460 rpm;
- Adding curing agent to the resin mixed with the filler in appropriate amounts;
- Mechanical mixing of the components of the composition at a speed of 460 rpm for a period of 3 min;
- Venting the epoxy composition over a period of 3 min.

For unmodified compositions, the step of introducing the filler into the resin was omitted.

Weighing of the components of the epoxy compositions was performed using a KERN CKE 3600-2 laboratory balance (Kern, Albstadt, Germany) with a measurement accuracy of 0.01 g.

For practical and metrological reasons, strength tests were carried out on model specimens with controlled geometries (prisms and rollers), conforming to PN-EN ISO standards. This approach allows an unambiguous assessment of the influence of the type of epoxy resin, curing agent, and filler on mechanical properties, while eliminating variables related to the type of substrate, uneven application, or film thickness. The resulting data reflect the actual material parameters and are widely used in the literature as authoritative predictors of functional coating behavior under operating conditions [28,29,30].

**Figure 3:**
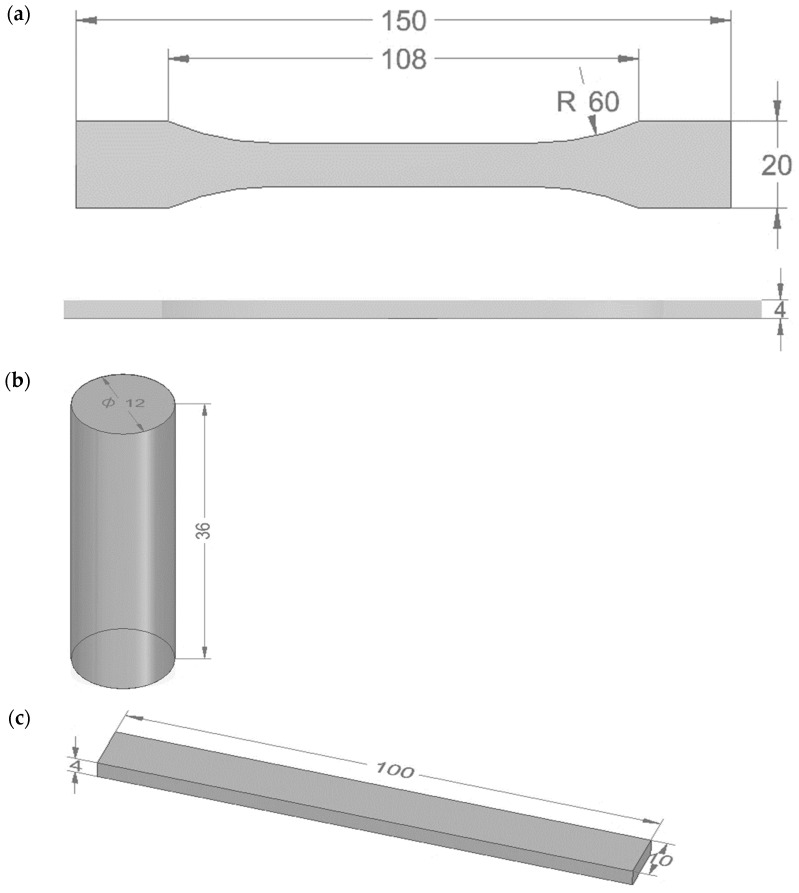
Shape and dimensions of the sample of epoxy compositions for testing (**a**) tensile strength (according to PN EN ISO 527-2 standard) [31], (**b**) compressive strength (according to ISO 604 standard) [32], and (**c**) bending strength (according to ISO 178:2003 standard) [33].

The compositions were then vented in a vacuum chamber to eliminate air bubbles. Venting was carried out using a CPS model VP6D two-stage vacuum pump (CPS, Florida, USA). The finished mixtures were poured into silicone molds, conforming to the dimensions of the samples specified in the applicable PN-EN ISO standards. The dimensions and geometry of the prepared samples are shown in Figure 3.

After one-step curing under laboratory conditions, the samples were seasoned for seven days invariably at 23 ± 2 °C at 23 ± 3% humidity to stabilize the network structure. After this time, strength tests were carried out and SEM photos were taken.

A series of 5 samples was prepared for each adhesive composition.

### 3.3. Material Testing

During the research, the strength properties of the epoxy compositions were tested. Tensile strength, compressive strength, and bending strength were tested and their structure was analyzed using scanning electron microscopy.

Tensile tests of epoxy compositions were carried out on a Zwick Roell Z150 testing machine (Zwick/Roell, Wroclaw, Poland) in accordance with PN EN ISO 527-1 (Plastics—Determination of mechanical properties in static tension) [31]. The crosshead traverse speed during the test was 5 mm/min, and the tensile modulus test speed was 1 mm/min. The initial test force was 30 N.

Compressive strength tests of the epoxy compositions were also performed on a Zwick Roell Z150 testing machine (Zwick/Roell, Wroclaw, Poland). These tests were carried out in accordance with ISO 604 (Plastics—Determination of Compressive Properties). This standard describes a method for determining, under certain conditions, the compression properties of plastics [32]. The assumed crosshead traverse speed during the test was 10 mm/min, and the compression modulus test speed was 1 mm/min. The initial test force was 20 N.

Bending strength tests were performed on a Zwick Roell Z2.5 testing machine (Zwick/Roell, Wroclaw, Poland) in accordance with DIN-EN ISO 178 (Plastics—determination of bending properties). This standard refers to a freely supported beam loaded at mid-span, i.e., three-point bending [33]. The test speed was 10 mm/min, and the initial test force was 5 N.

Microscopic examination of epoxy composition samples was studied using a TESCAN MIRA 3 GMX SE scanning electron microscope (SEM) (Tescan Orsay Holding, Brno-Kohoutovice, Czech Republic). The accelerating voltage was 10 kV.

### 3.4. Machine Learning Approach

This study employed a machine learning approach to support the prediction and analysis of the mechanical properties of epoxy adhesive compositions. Several supervised learning algorithms were tested to identify the most effective predictive models for tensile strength, compressive strength, and bending strength. The initial modeling phase involved developing and comparing multiple machine learning methods, including linear regression, support vector machines (SVMs), decision trees, random forests, and artificial neural networks (ANNs). Each algorithm was trained and validated using experimental data, including information on resin type, curing agent, filler type and concentration, and corresponding mechanical strength results.

For each model type, a range of hyperparameters were explored using grid search and cross-validation techniques to optimize performance. For neural networks, variations in the number of layers, the number of neurons per layer, and the activation functions (e.g., ReLU) were tested, as well as different regularization strengths. For SVM models, kernel types (linear, polynomial, and radial basis function (RBF)) and regularization parameters (C and gamma) were tuned.

While various algorithms were investigated, the study focuses on the models that performed the best prediction for each mechanical property. Specifically, neural network models achieved the highest accuracy in predicting tensile and bending strength, whereas the SVM model was most effective in predicting compression strength.

Support vector machine (SVM) is a supervised learning algorithm that constructs an optimal hyperplane to separate data points in a high-dimensional space. In regression tasks (SVR), the objective is to identify a function that differs from the actual target values by no more than a specified margin (ε), while maintaining the flattest possible model. SVMs are particularly effective at capturing non-linear relationships by using kernel functions (e.g., radial basis functions), which project input data into higher-dimensional feature spaces. In this study, the SVM model produced the most accurate predictions for compression strength, demonstrating its effectiveness in datasets involving complex and context-dependent interactions between variables [34,35].

Artificial neural networks (ANNs) are computational models inspired by the structure of the human brain. They consist of layers of interconnected nodes (neurons) that transform input data via weighted connections and activation functions. ANNs are well suited to modeling nonlinear relationships and capturing patterns in large, complex datasets. In this research, neural networks with different architectures (two and three hidden layers) and ReLU activation were used [34,35].

These models were selected based on the different indicators: $R^{2}$, the mean absolute error (MAE), the mean absolute percentage error (MAPE), the mean squared error (MSE), and the root mean squared error (RMSE) [34,35,36].

The coefficient of determination ($R^{2}$) shows what proportion of the variation of the dependent variable is explained by the model, and we estimate as follows:

(1) $$R^{2}=1-\frac{\sum_{i=1}^{k}{\left(y_{i}-{\hat{y}}_{i}\right)}^{2}}{\sum_{i=1}^{k}{\left(y_{i}-\bar{y}\right)}^{2}}$$

where $\bar{y}=\frac{1}{k}\sum_{i=1}^{k}y_{i}$.

The mean absolute error (MAE) quantifies the average magnitude of errors between predicted and actual values, measured using the $l_{1}$ norm. It is computed using the following formula:

(2) $$MAE=\frac{1}{k}\sum_{i=1}^{k}|y_{i}-{\hat{y}}_{i}|$$

The mean absolute percentage error (MAPE) quantifies the average magnitude of absolute errors as a percentage of the actual values and is computed using the following formula:

(3) $$MAPE=\frac{1}{k}\sum_{i=1}^{k}\frac{\left|y_{i}-{\hat{y}}_{i}\right|}{max\left(\epsilon ,|y_{i}|\right)}$$

where ϵ>0 is a small positive constant introduced to prevent undefined behavior when y is zero.

The Mean Squared Error (MSE) quantifies the average of the squared differences between predicted and actual values, serving as a standard metric for evaluating model accuracy. It is computed using the following formula:

(4) $$MSE=\frac{1}{k}\sum_{i=1}^{k}{\left(y_{i}-{\hat{y}}_{i}\right)}^{2}$$

The root mean squared error (RMSE) quantifies the typical magnitude of error by measuring the average deviation between predicted and actual values.

(5) $$RMSE=\sqrt{\frac{1}{k}\sum_{i=1}^{k}{\left(y_{i}-{\hat{y}}_{i}\right)}^{2}}$$

The final results and the interpretation of feature importance, including Shapley analysis, were based exclusively on these top-performing models [37]. This methodological approach ensured both model transparency and relevance to the physical behavior of the tested materials.

## 4. Results and Discussion

In this chapter, the obtained results of the strength tests will be presented, as well as the analysis of the taken SEM images of the breakthroughs of the samples.

### 4.1. Strength Test Results

Epoxy compositions used as protective layers—regardless of the substrate (metallic, concrete, or composite)—must meet several key conditions: provide adhesion, resistance to cracking and deformation, and impermeability to aggressive agents. Therefore, mechanical analysis, including tension, bending, and compression, provides information not only on structural strength but also on the material’s behavior as a functional barrier under operating conditions. The purpose of the strength study was to determine the effect of epoxy resin type, curing agent type, and filler grade and concentration on the mechanical properties of epoxy compositions that can act as protective coatings.

**Figure 4:**
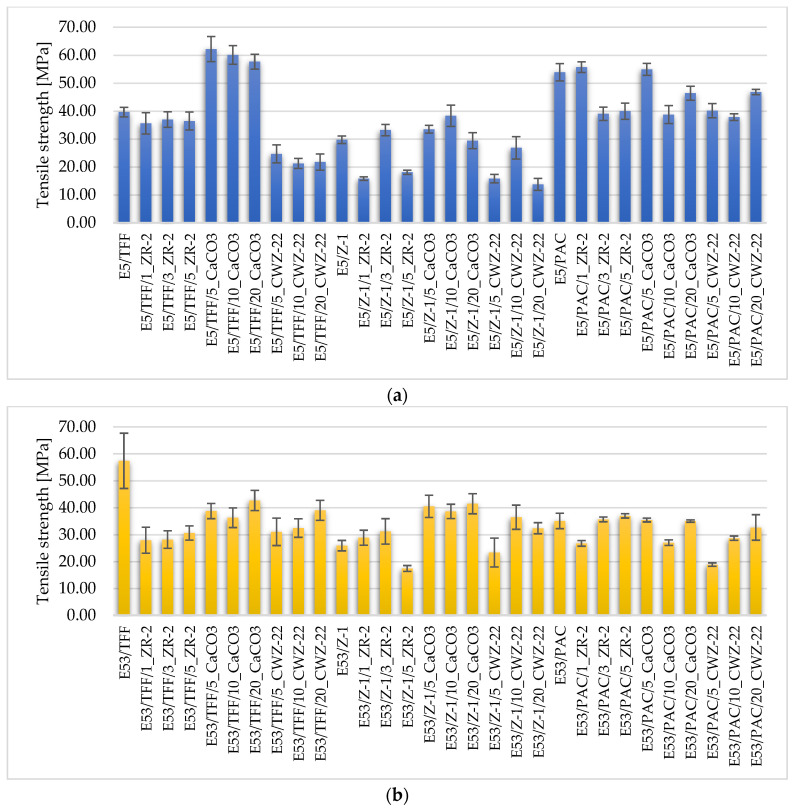

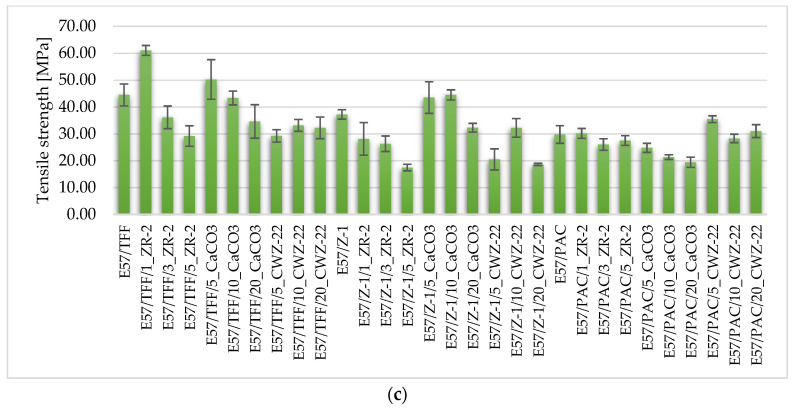
Tensile strength of epoxy compositions (**a**) based on Epidian 5 epoxy resin, (**b**) based on Epidian 53 epoxy resin, and (**c**) based on Epidian 57 epoxy resin.

**Figure 5:**
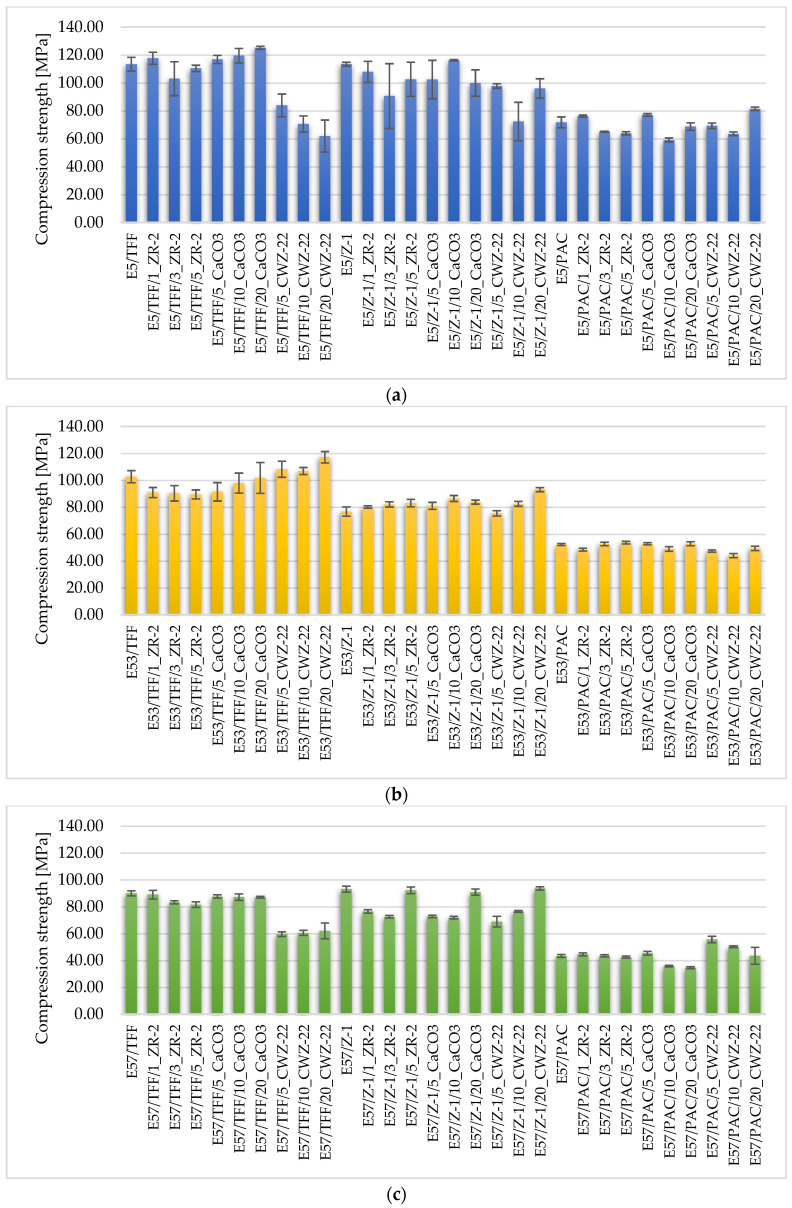
Compressive strength of epoxy compositions (**a**) based on Epidian 5 epoxy resin, (**b**) based on Epidian 53 epoxy resin, and (**c**) based on Epidian 57 epoxy resin.

**Figure 6:**
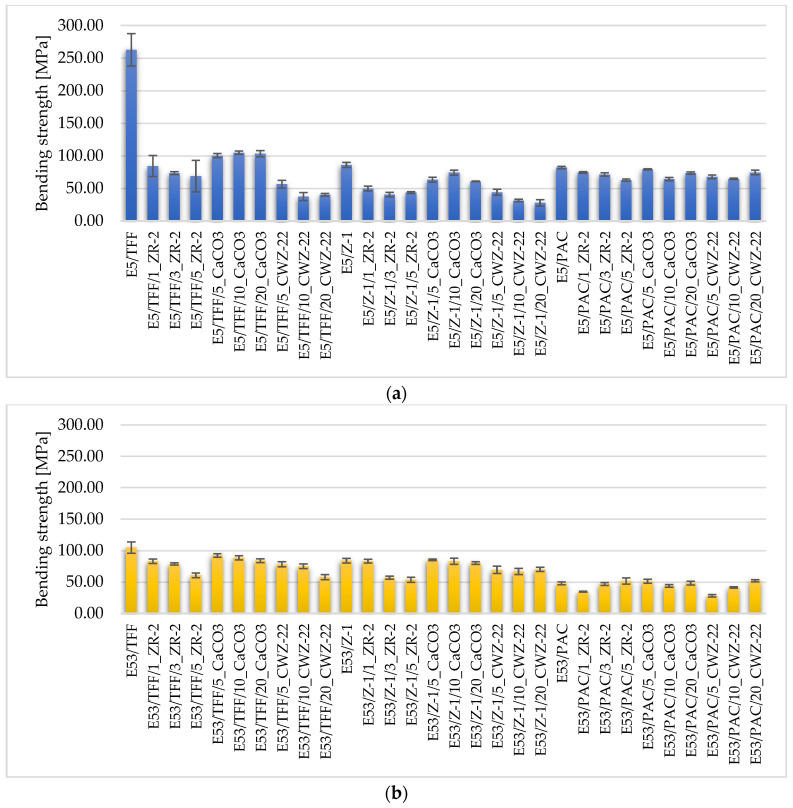

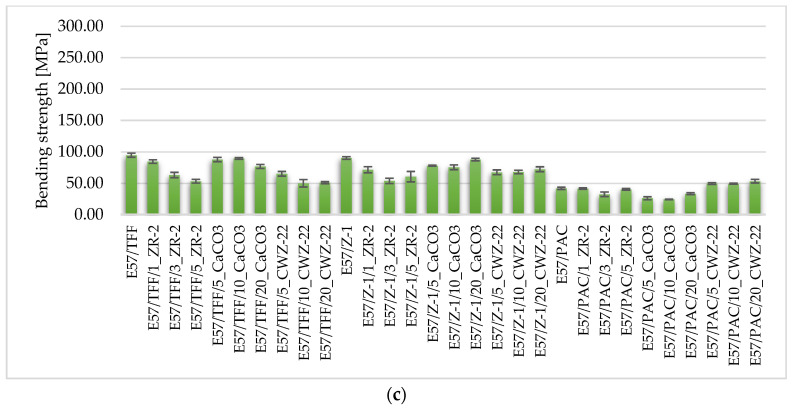
Bending strength of epoxy compositions (**a**) based on Epidian 5 epoxy resin, (**b**) based on Epidian 53 epoxy resin, and (**c**) based on Epidian 57 epoxy resin.

The mechanical properties of the tested epoxy compositions are shown in Figure 4, Figure 5 and Figure 6.

Figure 4 shows the tensile strength of the epoxy compositions. The best parameters were achieved for compositions containing Epidian 5 resin and TFF curing agent, especially in the presence of 10–20 wt% calcium carbonate (CaCO_3_). Tensile strengths exceeding 60 MPa in these compositions indicate effective stress transfer between the matrix and filler phases, which agrees with the results of Nwoye et al. [38], who confirmed an increase in mechanical resistance as a result of CaCO_3_ in epoxy systems.

In the case of compositions containing active fillers (CWZ-22), despite the good dispersion observed in SEM analysis, lower strength values were obtained. This indicates that activated carbons, despite their developed specific surface area and favorable contact with the matrix [39,40,41], do not provide mechanical strengthening of the structure. Their effect is mainly limited to improving functional properties such as thermal conductivity or resistance to chemicals.

Compositions with montmorillonite nanofiller ZR-2 showed a different behavior. At low concentrations (1–3%), especially in flexible matrices (e.g., E57/PAC), a moderate increase in strength was observed, which can be explained by better dispersion of the particles in the resin structure. This phenomenon has also been described by Foroutani et al. [13], indicating a relationship between mechanical properties and dispersion quality of nano-aluminosilicates. For higher concentrations of ZR-2 (5%), however, the effects were less favorable—agglomeration dominated, causing local weakening.

Compression test results (Figure 5) confirmed the high efficiency of the mineral reinforcement. The highest values—reaching up to 145 MPa—were obtained for compositions E5/TFF/20CaCO_3_ and E53/Z1/20CaCO_3_. The presence of rigid CaCO_3_ particles largely reduced local deformation, promoting the transfer of loads in a distributed manner, which corresponds to the classical model of passive reinforcement [42,43]. Importantly, these compositions, despite their higher stiffness, showed no signs of brittleness, thanks to the favorable configuration of interfaces and filler distribution in the matrix.

Fillers in the form of activated carbon (CWZ-22) did not improve compressive strength. Moreover, for compositions containing 20% CWZ-22 in harder resins (e.g., E53/Z1), a decrease in mechanical resistance was observed. This may be due to the presence of micropores around the carbon particles, visible in the SEM, and the fact that CWZ-22 activated carbon is not a structurally load-bearing filler but a functional modifier [13,44].

In the case of montmorillonite ZR-2, mixed results were evident: at low concentrations and in flexible compositions (E57/PAC), mechanical properties improved slightly, while in rigid matrices or at 5% addition, these effects were reversed. ZR-2 agglomeration and poor adhesion to the matrix were the main factors limiting the reinforcement efficiency, which correlates with data from Yu [45] and Yang et al. [42].

Bending, as a composite test, proved to be particularly sensitive to the quality of the interfacial bond and the uniformity of filler distribution (Figure 6). The highest bending values—exceeding 120 MPa—were obtained for compositions with CaCO_3_ in E5/TFF/10% and E53/Z1/20% compositions. These results indicate that the propagation of microcracks in the tensile zone is effectively limited and the structural integrity of the entire coating is high. Fu and co-authors [46] note that well-distributed mineral particles can act as crack traps, delaying crack propagation.

**Figure 7:**
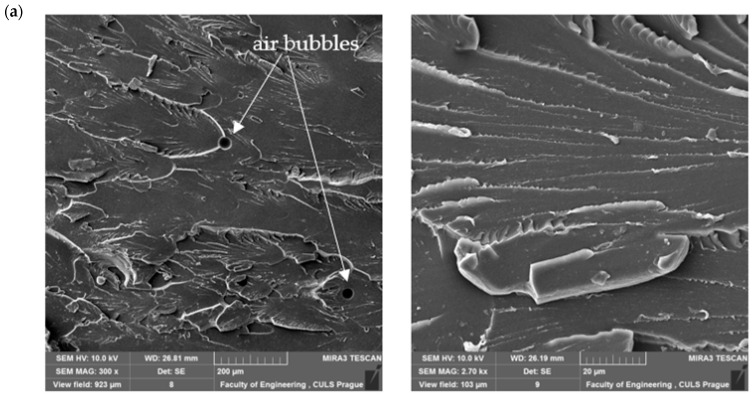

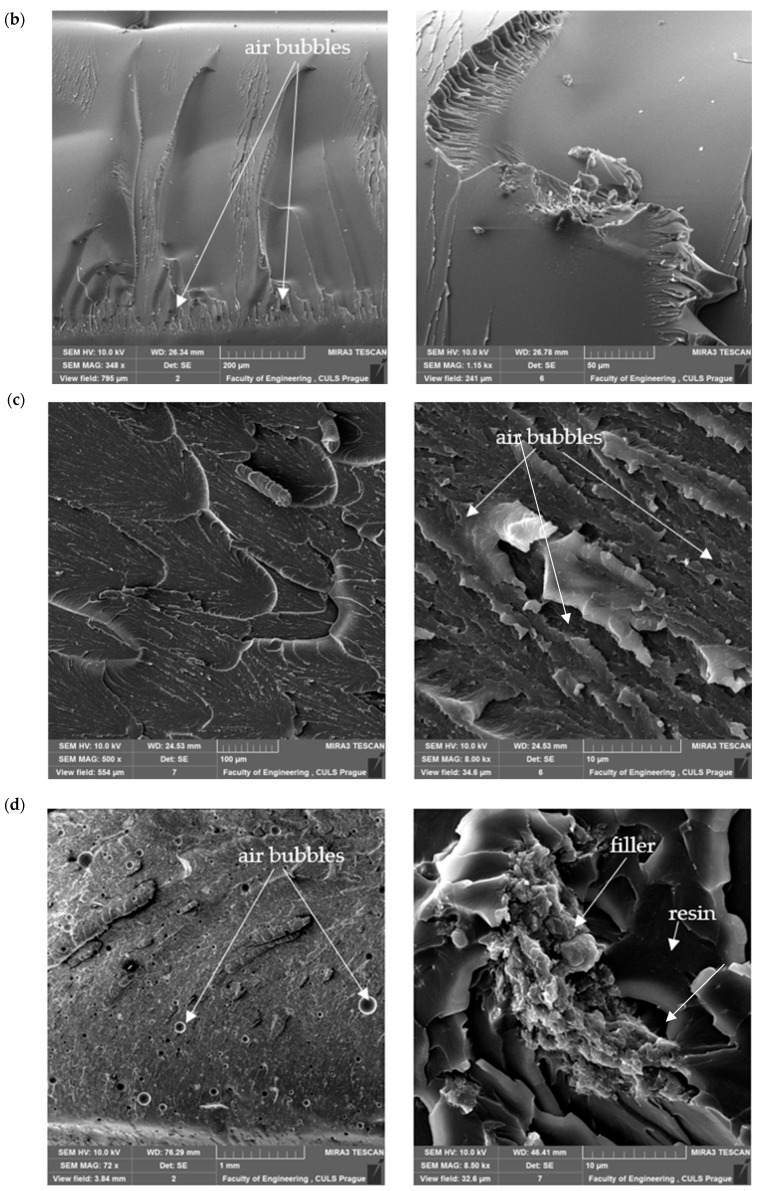

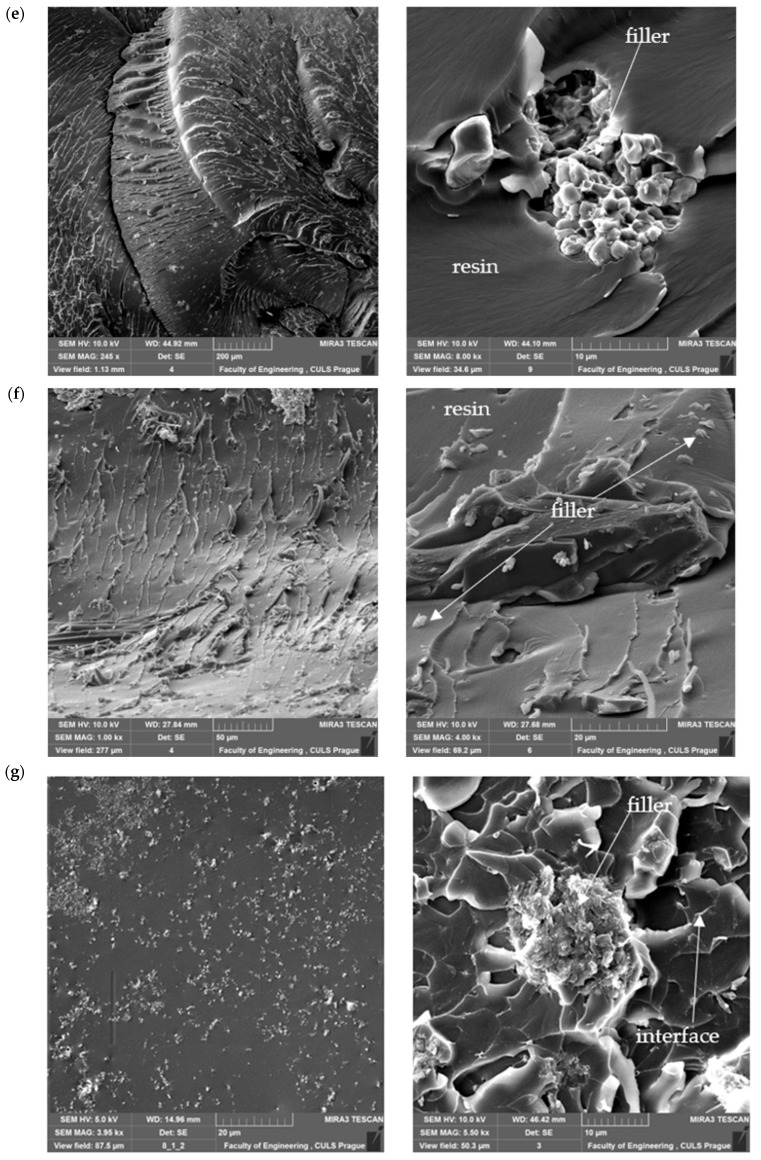

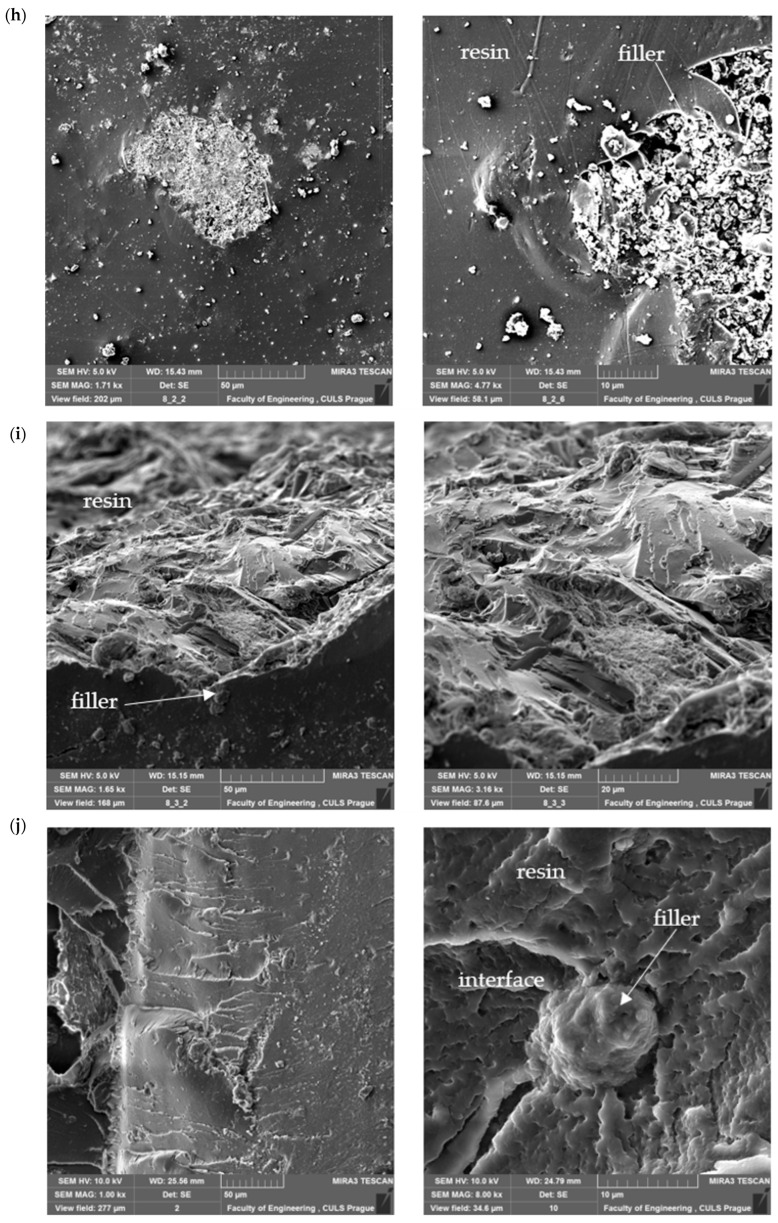

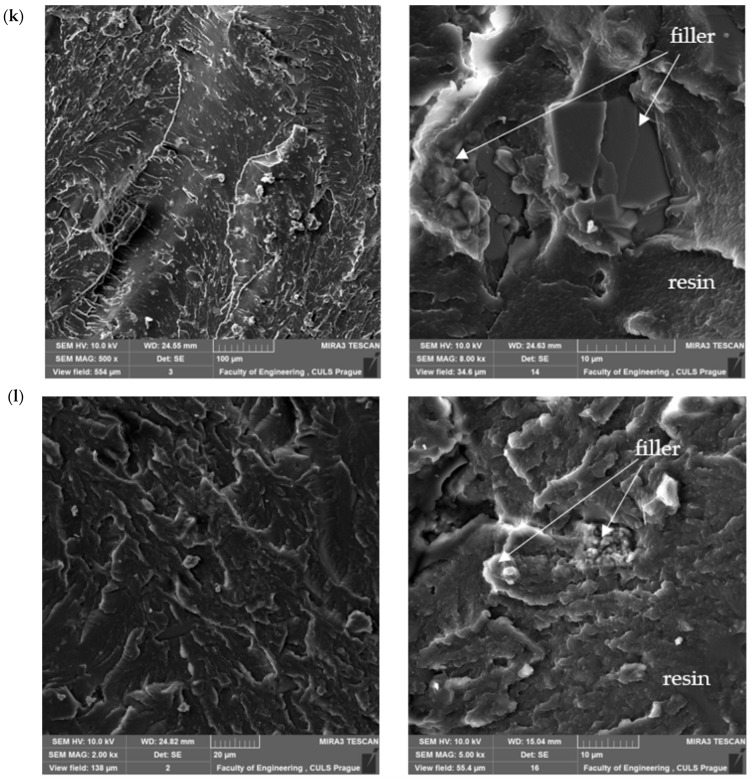
SEM images of selected epoxy compositions: (**a**) E57/TFF; (**b**) E57/Z1; (**c**) E57/PAC; (**d**) E5/TFF/5ZR-2; (**e**) E5/TFF/20CaCO_3_; (**f**) E5/TFF/20CWZ-22; (**g**) E53/Z1/5ZR-2; (**h**) E53/Z1/20CaCO_3_; (**i**) E53/Z1/20CWZ-22; (**j**) E57/PAC/5ZR-2; (**k**) E57/PAC/20CaCO_3_; (**l**) E57/PAC/20CWZ-22.

Compositions containing CWZ-22, even with good wettability (Figure 7l), showed no increase in flexural strength. The presence of local agglomerates and micropores, also observed in SEM analysis, indicates that good dispersion does not guarantee mechanical strengthening—in line with the observations of Yang and Heo [42].

In compositions with montmorillonite, the improvement in flexural properties was limited to compositions with a flexible matrix (E57/PAC). In rigid compositions, no strengthening effect was observed—moreover, at higher ZR-2 concentrations, weakening effects related to phase discontinuity were evident.

### 4.2. SEM Analysis of Microstructure

In order to further analyze the influence of material composition on the functional properties of epoxy coatings, SEM microscopic observations of selected compositions were carried out. A set of twelve compositions (Figure 7) was selected so that the microstructure of materials differing in both the type of epoxy resin (E5, E53, E57) and the type of curing agent (TFF, Z1, PAC) and the filler used (ZR-2, CaCO_3_, CWZ-22) could be compared. The obtained images made it possible to identify differences in the dispersion of the dispersed phase, the quality of interfacial adhesion, and the presence of defects, which can have a key impact on the durability and effectiveness of protective coatings made from the described compositions.

An image of a reference sample of the E57/TFF composition (Figure 7a), containing only resin and curing agent, shows a homogeneous, continuous epoxy matrix, with visible individual pores (gas bubbles), without microcracks or delamination zones. This structure confirms the correct course of crosslinking and provides a reference point for evaluating the influence of modifying additives. In comparison, a sample of the E57/Z1 composition (Figure 7b), where the combination of the E57 resin with the stiff Z1 curing agent led to a morphology with significantly higher stiffness. The SEM microscopic image reveals the presence of microcracks and sharp phase transitions, typical of compositions with a high degree of cross-linking and less ability to relax stress. Such structures may be prone to brittleness, limiting their effectiveness as protective coatings in dynamic environments. Different structural properties were observed in a sample of the E57/PAC composition (Figure 7c), which uses the same type of resin but a more flexible curing agent, showing a slight waviness of the matrix surface, which may indicate a reduced degree of cross-linking and greater susceptibility to plastic deformation. The lack of visible defects confirms the good structural uniformity of the flexible compositions.

The addition of fillers introduces significant morphological changes. The E5/TFF/5ZR-2 composition sample (Figure 7d), which contains a low weight share of montmorillonite, shows the presence of small, lamellar ZR-2 particles. Their dispersion is moderate—locally there are agglomerates and small zones of stratification, which may limit the efficiency of stress transfer and affect local porosity. In addition, gas bubbles are visible, occurring on the surface of the tested breakthrough of the composition sample. In contrast, E57/PAC/5ZR-2 (Figure 7j), despite the identical filler concentration, presents a much more homogeneous structure with better particle embedding in the elastic matrix. The absence of defects and the correct dispersion testify to the synergy of the elastic matrix and the well-dispersed nano phase, which may explain the enhanced dynamic properties of this composition.

Significant differences are also observed when comparing CaCO_3_ filled structures. The E5/TFF/20CaCO_3_ sample (Figure 7e) is characterized by a compact structure with well-submerged filler particles, which is conducive to increased compressive strength. At the same time, the lack of local cracks and voids may indicate effective load transfer. However, agglomerations of filler particles are noticeable. In contrast, in the E53/Z1/20CaCO_3_ sample (Figure 7h), a tighter packing of the filler in a rigid matrix was observed, with the presence of micropores and discontinuities at the phase boundaries. This configuration promotes stress concentration and may increase the coating’s susceptibility to crack initiation.

In compositions containing CWZ-22 activated carbon in particulate form, such as E5/TFF/20CWZ-22 (Figure 7f), E53/Z1/20CWZ-22 (Figure 7i), and E57/PAC/CWZ-22 (Figure 7l), significant inhomogeneity in the distribution of the filling phase is noticeable. Activated carbon particles form irregularly shaped agglomerates, around which microcracks and porosity are often present. Particularly evident is poor adhesion between the filler and matrix, which limits structural integrity and can reduce barrier properties and resistance to service loads. In the case of sample E53/Z1/20CWZ-22, pronounced defects appear at the phase boundaries, indicating the possibility of delamination under varying loads. This situation may be due to the high proportion of filler in the composition.

The E53/Z1/5ZR-2 epoxy composition (Figure 7g), containing a low proportion of nanofiller in the solid resin, is characterized by the presence of zones of delamination and incomplete phase integration. Although particle aggregation is less severe than in the case of the CWZ-22 filler, the presence of voids and irregular phase transitions can negatively affect mechanical performance, according to the results of tensile and bending tests.

Finally, in the E57/PAC/20CaCO_3_ sample (Figure 7k), a compact structure with clearly marked clusters of mineral filler is noted. Although agglomerates are not present, single cracks were observed, indicating correct compatibility. Local stiffening may limit deformability and adversely affect coating behavior in dynamic environments.

A compilation of SEM images proves that the microstructural properties of epoxy composites significantly depend on both the type of filler and flexibility of the matrix and the quality of interfacial interactions. Compositions characterized by structural homogeneity and good adhesion show better mechanical properties and protection potential. In contrast, the presence of agglomerates, microcracks, and delamination, typical of active fillers and rigid compositions, can significantly limit the functionality of the material in engineering applications.

## 5. Prediction Results Analysis

Different machine learning methods were applied, including neural networks and support vector machines, to develop models for three essential mechanical properties of the material: tensile strength (TS), compression strength (CS), and bending strength (BS). Each method was selected to ensure that the model architecture was appropriately suited to the specific characteristics and predictive challenges of the target property.

Table 10 shows the hyperparameters and structural details of the machine learning models that were most effective at predicting the TS, CS, and BS of the tested material. Each property was modeled using a different machine learning technique that was carefully selected based on its performance during training and validation. The table includes the following relevant configuration parameters: model architecture, activation function, regularization settings, training time, prediction speed, and data pre-processing methods.

For the TS, the bilayered NN model was the best. This model employed a simple feedforward architecture consisting of two hidden layers, each with ten neurons (layer sizes: 10–10). Both layers used the ReLU (rectified linear unit) activation function, which is commonly used due to its efficiency and ability to handle nonlinear relationships. No regularization was applied (λ = 0), and the input data were standardized prior to training. Training the model took approximately 2.59 s, and it offers a prediction speed of 73,000 observations per second. While the model is computationally efficient, its predictive accuracy for tensile strength was relatively low, suggesting that further refinement or architectural adjustments may be necessary.

The best results for CS were obtained using an SVM (support vector machine) model. As SVMs are not neural networks, parameters such as layers and activation functions are not applicable in this context. The regularization setting was configured as ‘Auto’, enabling the model to adaptively balance complexity and generalization. As with the other models, the input data were standardized. Training took approximately 2.62 s, with the model achieving a prediction speed of 23,000 observations per second. While this is slower than neural network models, it is still acceptable given the high level of accuracy. This model demonstrated excellent performance, proving highly effective in predicting compression strength.

The model for BS was also modeled using a neural network, which proved to be the most successful model in terms of predictive accuracy overall. This network was deeper, consisting of three hidden layers, each with ten neurons (layer sizes: 10–10–10). As with the tensile model, the ReLU activation function was used throughout. No regularization was applied (λ = 0), and the data were standardized prior to training. Training this model took only 1.51 s—the shortest time of all three models—and it achieved a prediction speed of 67,000 observations per second. This makes it suitable for fast, scalable applications. The combination of a deeper structure and an efficient activation function enabled this model to accurately capture the complex relationships governing bending strength.

Model performance was evaluated using standard indicators (Equations (1)–(5)) and the interpretation of feature importance via Shapley values. This enabled an assessment of both overall predictive accuracy and the contribution and influence of individual input variables in the prediction process. Model selection was based on a combination of multiple evaluation metrics, including R^2^, RMSE, MAE, and MAPE. Although R^2^ and RMSE were prioritized due to their intuitive interpretability and widespread use in materials research, all four metrics were considered in order to evaluate both absolute and relative accuracy. No formal weighting scheme was applied; instead, a comparative approach was used to select the model offering the most balanced and consistent performance across these indicators.

In the case of tensile strength (TS), the selected neural network model achieved the highest R^2^ among all tested configurations, but its high MAPE indicated low reliability. This limitation was acknowledged, and the model was retained not for prediction in practice but to identify possible future directions for feature engineering and data refinement. As such, its feature importance results are interpreted cautiously and framed as exploratory insights.

Table 11 shows the results of the most successful predictive models developed to estimate three important mechanical properties of materials: tensile strength, compressive strength, and bending strength. Various machine learning techniques, including NN and SVM, were applied to each property during the modeling process. The method that achieved the highest accuracy during training and validation was selected for each property, and only these models are included in the table. The table summarizes the performance of these models based on the following key validation metrics: R-squared (R^2^), root mean square error (RMSE), mean square error (MSE), mean absolute error (MAE), and mean absolute percentage error (MAPE).

Based on the presented results, the NN model for predicting BS achieved the highest accuracy, with an R^2^ value of 0.95. This indicates an excellent alignment between the predicted and actual values. Similarly, the SVM model for CS achieved an R^2^ value of 0.93, also demonstrating strong predictive performance. These models also reported very low error values across all remaining metrics—for example, RMSE values of around 6.4–6.6 MPa, MAE below 5 MPa, and MAPE values below 7%—making them highly reliable for practical applications such as quality control, simulation, and materials optimization.

Conversely, the NN model for TS performed considerably worse. It achieved a relatively low R^2^ value of 0.64, suggesting that a significant proportion of the variance in the target variable remains unexplained. It also produced substantially higher error values: an RMSE of 4483.5, an MAE of 694.66, and an exceptionally high MAPE of 1494.2%. This indicates that its predictions are highly inaccurate and unreliable.

Several steps were undertaken in an attempt to improve the predictive performance of the TS model. These included optimization of ANN hyperparameters (e.g., number of layers and neurons, activation function types such as ReLU and Tanh, and regularization techniques), as well as testing of alternative algorithms such as support vector regression (SVR), decision trees, random forests, and ridge regression. In addition, various data preprocessing techniques, including standardization, min-max scaling, and logarithmic transformations, were evaluated. Despite these efforts, the TS model consistently underperformed in comparison to models for compression and bending strength. This suggests that the tensile strength data may be influenced by additional latent factors—such as filler–matrix interfacial variability, dispersion quality, or localized defects—which are not sufficiently captured by the current input features.

As a result, the interpretation of Shapley feature importance in the TS model should be regarded as indicative rather than conclusive. We caution against drawing definitive conclusions about the dominant predictors of tensile strength based on this model alone.

Due to the models’ predictive performance in relation to TS, CS and BS, a deeper analysis was conducted to better understand the factors contributing to their accuracy. Advanced interpretability techniques based on Shapley values were employed to accomplish this, including Shapley importance and Shapley summary plots. These tools enable transparent evaluation of each model’s decision-making process by quantifying the contribution of each input feature to the model’s output.

The Shapley approach, which is based on cooperative game theory, calculates the Shapley value for each feature, representing the average marginal contribution of that feature across all possible subsets of input variables. This method ensures fair and balanced influence attribution, as it considers every possible feature combination and weighs each contribution equally. Applying this analysis to the best-performing models for each mechanical property provides a detailed picture of how different features influence predictions both globally (across the dataset) and locally (per observation).

The Shapley analysis not only reveals which variables have the greatest impact on model predictions but also the nature and direction of that impact. For instance, some features may consistently increase predicted values, while the effect of others depends on the input configuration, either increasing or decreasing predictions. This level of interpretability is crucial for validating model behavior, identifying key design parameters and supporting informed decisions in material optimization.

**Figure 8:**
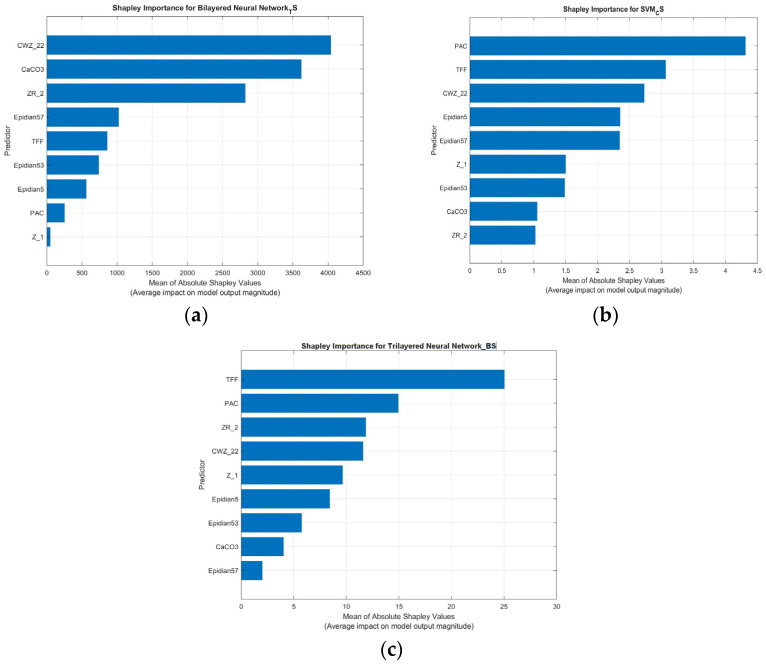
Shapley importance plots for the developed models: (**a**) NN model for TS; (**b**) SVM model for CS; (**c**) NN model for BS.

**Figure 9:**
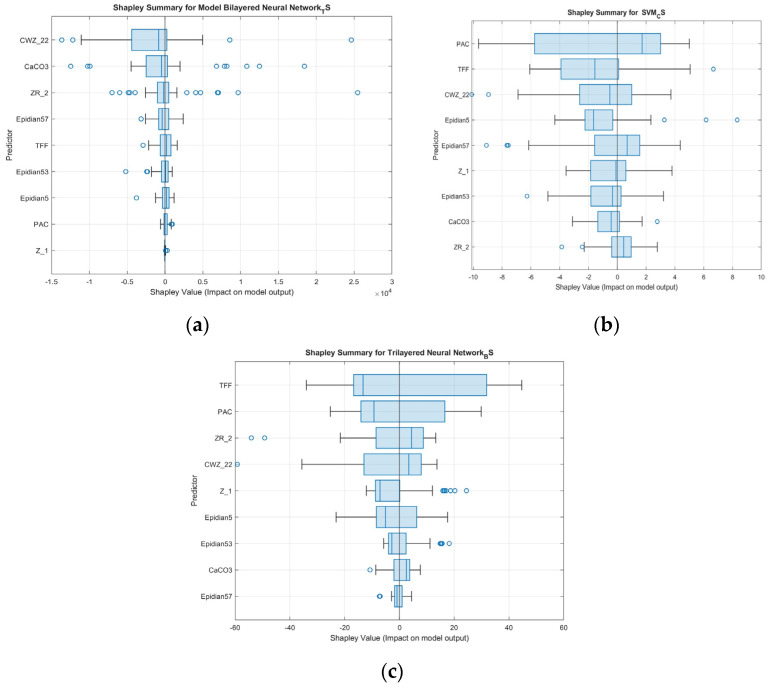
Shapley summary plots for the developed models: (**a**) NN model for TS; (**b**) SVM model for CS; (**c**) NN model for BS.

Figure 8 and Figure 9 present a set of Shapley plots, providing a detailed explanation of how input features influence the prediction of three key mechanical properties: tensile strength, compression strength, and bending strength. These plots include both importance and summary plots.

The plots indicate the average absolute impact of each feature on model predictions. In the TS model, CWZ-22 had the highest importance with a maximum Shapley value of 4040.02, while Z-1 had the lowest at 51.16. For CS, PAC dominated with 8.57, and Z-2 contributed the least with 0.69. In the BS model, TFF had the strongest influence (41.17), while CaCO_3_ showed the lowest (4.26). These values support the identification of the most influential formulation variables across each property.

The plots (Figure 9a–c) show both the magnitude and direction of the effect each feature has on individual model predictions. For TS, CWZ-22 exhibits a wide spread of Shapley values (both positive and negative), suggesting its contribution varies depending on specific formulation values. In contrast, Z-1 has consistently low influence.

In the CS model, PAC shows strong and mostly positive influence, while Z-2 has limited effect. For BS, TFF consistently contributes positively to predicted strength, whereas CaCO_3_ has a minor and slightly varying influence.

The Shapley importance plot in the TS model (Figure 8a) indicates that the most significant predictors are CWZ-22, CaCO_3_, and ZR-2. These three variables clearly have the highest mean absolute Shapley values, confirming that they dominate the model’s predictions. Their physical roles may relate to matrix stiffening (CWZ-22), filler content (CaCO_3_), and reinforcement or fiber content (ZR-2), all of which typically have a significant impact in tensile loading conditions.

The Shapley summary plot (Figure 9a) further clarifies how these variables influence individual predictions. The widespread Shapley values for CWZ-22 suggest that, although it is generally impactful, its contribution can vary considerably from sample to sample. This indicates some level of model instability or nonlinearity in how this variable interacts with others. The same is true, albeit to a lesser extent, of CaCO_3_ and ZR-2.

Secondary variables such as Epidian 57, TFF, and Epidian 53 demonstrate modest and relatively narrow distributions, with Shapley values centered around zero. Their role is more supportive and consistent. Variables such as PAC, Epidian 5, and, in particular, Z-1 demonstrate negligible importance and minimal variance, suggesting that they are not relevant for predicting tensile strength.

Despite identifying several key drivers, the model’s overall predictive performance remains relatively low (R^2^ = 0.64). This may be due to overreliance on a few strong predictors and the shallow depth of the network (two hidden layers).

The model that predicts CS has a notably different importance profile (Figure 8b). The Shapley importance plot identifies PAC, TFF, and CWZ-22 as the top contributors. This is in contrast to the tensile strength model, where PAC was among the least relevant features. Here, however, PAC dominates, achieving the highest average Shapley value, closely followed by TFF.

The Shapley summary plot (Figure 9b) provides more nuance. The wide box plot for PAC, extending significantly in both positive and negative directions, shows that PAC can have both enhancing and suppressing effects on the model output, depending on the context. This suggests that the model has learned conditional relationships, for which SVMs operate well due to their ability to define non-linear boundaries. TFF shows similar bidirectional behavior, albeit with slightly less variability. Although influential, CWZ-22 has a more constrained and generally positive distribution.

Other variables, such as Epidian 5, Epidian 57, and Z1, demonstrate moderate importance with some variability. Conversely, CaCO_3_ and ZR-2—highly important in the tensile model—have little influence here, highlighting the property-specific relevance of features.

This SVM model achieves excellent predictive accuracy (R^2^ = 0.93) due to its ability to leverage complex, context-sensitive variables effectively, particularly PAC and TFF.

In the BS prediction task, the Shapley importance plot (Figure 8c) identifies TFF as the most critical feature, followed by PAC, ZR-2, and CWZ-22. These variables are key contributors to the model’s high accuracy (R^2^ = 0.95) and robust generalization.

The summary plot shows (Figure 9c) that TFF consistently has a positive influence across samples, establishing it as a stable and dominant predictor. PAC and ZR-2 also demonstrate a strong and varied influence, which is sometimes positive and sometimes negative. This suggests that their impact is more nuanced and sample-dependent. CWZ-22 retains moderate importance, but its range of influence is narrower than that of tensile strength predictions.

The lower-ranked variables—Z1, Epidian 5, Epidian 53, CaCO_3_, and Epidian 57—contribute little to the model’s decisions, with low and narrow distributions of Shapley values. Their roles are minor, and they do not appear essential in the bending context.

The model provides sufficient depth and flexibility to capture a broad range of interactions and nonlinear relationships. This is evident in how it balances multiple important features without depending excessively on a single variable.

The combined analysis of the Shapley importance and summary plots provides several key conclusions:

- TFF is the only variable that is consistently important across all three models. It is particularly dominant in the bending strength model, where it has the highest and most stable positive average impact. This suggests that TFF plays a versatile role, influencing a range of mechanical properties, which is likely due to its structural or adhesive characteristics in the composite.
- PAC is highly influential for compression and bending strength but almost irrelevant for tensile strength. Its behavior is variable and sometimes bidirectional, indicating that its role depends heavily on its interaction with the other components of the formulation.
- CWZ-22 has the greatest impact on tensile strength prediction, with reduced relevance in compression and bending. This suggests that its contribution may relate to stiffness or network formation under tensile loads.
- ZR-2 is important for tensile and bending strength but not compression, mirroring the pattern observed for CWZ-22.
- CaCO_3_ is a key predictor only in the tensile model, highlighting its specific role in enhancing tensile resistance, likely through particle reinforcement or matrix modification.
- Z1, Epidian 53, Epidian 5, and Epidian 57 remain minor contributors across all tasks, though some exhibit small but variable influences, especially in specific samples.

Importantly, the Shapley summary plots emphasize the difference in impact directionality. The SVM model shows the greatest use of bidirectional contributions, especially for PAC and TFF, while the neural network models—particularly the deeper one used for bending—show more consistent, stable patterns of feature importance.

In addition to identifying key predictors, the Shapley analysis was interpreted within the context of materials science to determine why certain features dominate particular mechanical responses.

For example, PAC (porous activated carbon) was found to have the greatest influence on compression strength (CS). This is consistent with its fine particle size, rigidity, and ability to improve stress distribution and energy dissipation under compressive loading. In contrast, its impact on tensile strength (TS) was much lower, likely due to interfacial limitations—carbon-based fillers can act as stress concentrators if dispersion or adhesion is suboptimal.

Conversely, CWZ-22—a surface-modified filler—demonstrated the strongest influence on TS. Its enhanced surface activity likely improves interfacial bonding with the matrix, which is critical under tensile stress, as microcracks and failure typically initiate at the filler–matrix interface. However, its effect on CS and BS was lower, as these properties are more influenced by bulk behavior and particle morphology than by interfacial chemistry alone.

The widespread Shapley values observed for CWZ-22 also suggest that its contribution varies depending on other interacting features, such as concentration, resin type, or hardener system. This highlights the complexity of multi-phase composite behavior and the importance of analyzing not just main effects, but also interactions. This will be the subject of future work.

The trends identified through machine learning feature importance analysis were examined further in light of the microstructural observations obtained via SEM imaging (see Figure 7). Fillers exhibiting microporosity, weak interfacial bonding, or agglomeration, such as PAC and CaCO_3_, were associated with limited improvements or even reductions in mechanical strength, particularly in tensile and bending tests. While these effects were not explicitly included as input features in the ML models, they are likely to be reflected implicitly in the ‘filler type’ variable, which serves as a proxy for the filler’s physical behavior, dispersion quality, and interfacial characteristics.

For instance, the relatively low Shapley values of CaCO_3_ across all strength properties are consistent with SEM findings of filler clustering and resin detachment. In contrast, CWZ-22 showed a more homogeneous distribution and fewer voids, which is consistent with its strong influence on tensile strength in the Shapley analysis. These correlations suggest that microstructural defects may explain why certain fillers underperform despite their chemical potential and highlight the importance of linking data-driven model interpretation with physical evidence from microscopy.

This extensive analysis confirms that each mechanical property is governed by a unique set of predictors, and the same variable can have entirely different roles depending on the target. It also underscores the need for dedicated model architectures and feature importance evaluation when working with complex materials data.

By combining global (mean) and local (distributional) interpretability tools like Shapley values, we gain not only performance insights but also practical knowledge for material design. This can support targeted optimization, formulation tuning, and data-driven decision-making in engineering applications.

Although the proposed machine learning (ML)-based approach shows strong potential for modeling and interpreting the effects of formulation variables, it also has inherent limitations. The models are trained on a finite dataset and may lose predictive power when applied to formulations outside the composition space that has been explored. Furthermore, complex microstructural interactions that are not captured by the input variables (e.g., filler distribution or interfacial defects) may reduce the accuracy of the models.

Nevertheless, this method is particularly well-suited to polymer composite systems where multiple variables influence multiple target properties and exhaustive experimental campaigns are impractical. The method’s generalizability lies in its modular workflow: while specific models may require retraining for other systems, the combined use of targeted experimentation, machine learning modeling, and Shapley-based interpretation remains broadly transferable.

## 6. Conclusions

The conducted research clearly confirms the effectiveness of physical modification of epoxy adhesive materials using mineral, active, and nanofillers in the context of designing functional protective coatings. The results of mechanical tests and SEM microstructural analysis showed a significant influence of both the chemical composition and interfacial interactions on the behavior of the compositions under service loading conditions.

Among the tested systems, compositions based on Epidian 5 epoxy resin and TFF curing agent, modified with the addition of CaCO_3_ calcium carbonate (10–20 wt%), showed the highest strength parameters (more than 60 MPa in tension, 145 MPa in compression, and 120 MPa in bending). This confirms that classical mineral fillers, when properly dispersed, can effectively strengthen the composite structure and increase its resistance to degradation under mechanical conditions.

Active fillers, such as CWZ-22 activated carbon in particulate form, despite favorable performance characteristics (vibration damping, conductivity), did not contribute to improving strength parameters, as confirmed by both mechanical strength and microstructure analysis (presence of pores, poor phase adhesion). On the other side, the use of montmorillonite ZR-2 allowed for obtaining favorable effects only in compositions with an elastic matrix (e.g., E57/PAC), where the particles were better dispersed and integrated into the continuous phase.

The novelty of this paper was the successful combination of classical experimental approaches with data analysis tools and machine learning methods. Machine learning models, based on input data on the type of resin, curing agent, and filler concentration, enabled effective prediction of mechanical properties of composites. This approach significantly improves the design process of epoxy compositions, which can also be used as functional protective coatings, enabling the selection of optimal formulations without the need to conduct costly, labor-intensive, and time-consuming tests each time. Predictive models were developed to forecast the mechanical performance of compositions based on input data such as the type of resin, hardener and filler, and their respective concentrations. Three high-performance models were selected:

- For tensile strength, a 2-layer neural network model achieved an R^2^ = 0.64, an RMSE = 4483.5, and a MAPE = 1494.2%.
- For compression strength, an SVM model achieved an R^2^ = 0.93, an RMSE = 6.42, and a MAPE = 5.0%.
- The most accurate model for bending strength was a 3-layer neural network, which obtained an R^2^ = 0.95, an RMSE = 6.62, and a MAPE = 7.1%.

However, the prediction model for tensile strength demonstrated limited reliability, with a moderate R^2^ value (0.64) and high error metrics. Multiple approaches were tested to improve this model—including algorithm switching, parameter tuning, and data transformations—but without significant success. This limitation suggests that tensile strength may be influenced by more complex or microstructural factors not included in the current dataset. Therefore, the feature importance results from the TS model should be treated with caution and not considered as definitive evidence of factor influence. Future work should consider expanding the dataset or integrating SEM-based descriptors to improve TS prediction accuracy.

Moreover, the results demonstrate that different modeling approaches are optimal depending on the nature of the mechanical property. Shapley value analysis enabled detailed interpretation of feature importance:

- TFF was the most consistent predictor across all models, particularly in bending.
- PAC significantly influenced compression and bending strength but had little effect on tensile strength.
- CWZ-22 and CaCO_3_ were key drivers of tensile strength.

This interpretability is crucial for validating model predictions and directing future formulation efforts.

The analysis confirms that the integrated approach—combining microstructural observations, strength analysis, and predictive modeling—is an effective methodology for designing modern engineering materials. The results obtained can be successfully applied in industrial practice, especially in areas requiring coatings with high mechanical and chemical resistance, such as the construction, energy, marine, and automotive industries.

However, some limitations of the study should be noted. Although the dataset was carefully designed, it may not fully capture the variability in resin types, filler geometries, or environmental conditions affecting long-term performance. Furthermore, the current models are based on static laboratory data and do not consider real-world degradation mechanisms, such as UV exposure, moisture absorption, or thermal cycling. While the ML models applied were effective, they were based on relatively standard architectures and did not exploit deeper learning methods or ensemble approaches to their fullest potential.

Future research should focus on integrating advanced machine learning models, such as random forests, XGBoost, and deep neural networks, to more accurately capture the non-linear interactions between the input features and the predicted mechanical properties. Due to their greater complexity and flexibility, these algorithms are well suited to modeling high-dimensional, heterogeneous material data.

Another important area for research is to extend experimental datasets to include information on durability and the effects of aging, such as the long-term impact of environmental factors (e.g., humidity, UV radiation, and temperature fluctuations). Incorporating this data would enable long-term performance to be predicted and would enhance the applicability of the models in real-world operating conditions.

In parallel, future studies should place greater emphasis on analyzing full-scale coatings, considering parameters such as actual coating thickness, porosity, and interfacial adhesion. These characteristics significantly influence the performance of coatings in industrial applications and must be accounted for to improve the practical relevance and reliability of predictive models.

Although the developed machine learning models initially require a representative set of experimental data, they provide a valuable tool for future material design. Once trained, they facilitate the rapid in silico screening of new formulations, thereby reducing the need for extensive physical testing. This can shorten development time and reduce costs, particularly when only the top performers are selected for validation. Furthermore, the model architecture and learned relationships can be adapted to similar epoxy systems, facilitating knowledge transfer across related projects.

Finally, considerable potential exists in developing hybrid experimental–computational workflows that combine machine learning predictions with finite element simulations or micromechanical modeling. These multiscale approaches can bridge the gap between the structure of materials at a microscopic level and their behavior at a macroscopic level, enabling a more comprehensive and physics-informed process for designing materials.

## Figures and Tables

**Table 1:** Physical and chemical properties of the epoxy resins used in the study [19,20,21,22,23].

Properties	Epidian 5 Epoxy Resin	Epidian 53 Epoxy Resin	Epidian 57 Epoxy Resin
Epoxy number	0.48–0.52 mol/100 g	≥0.41 mol/100 g	≥0.40 mol/100 g
pH value	ok. 7	ok. 7	ok. 7
Viscosity at 25 °C	20,000–30,000 mPa·s	900–1500 mPa·s	13,000–19,000 mPa·s
Density in 20 °C	1.16 g/cm^3^	1.11–1.15 g/cm^3^	1.14–1.17 g/cm^3^
Flash temperature	266 °C	58 °C	134 °C
Temperature auto-ignition	490 °C	460 °C	455 °C
Melting point	30–50 °C	Not applicable	Not applicable
Initial boiling point	not marked—distribution	141 °C	>215 °C does not boil

**Table 2:** Properties of the curing agents used in the tests [23,24].

Properties	Mannich’s Principle (TFF Curing Agent)	Amine Curing Agent (Z-1 Curing Agent)	Polyamide Curing Agent (PAC Curing Agent)
Viscosity at 25 °C	max. 10,000 mPa·s	20–30 mPa·s	10,000–25,000 mPa·s
Density in 20 °C	1.15–1.20 g/cm^3^	0.978–0.983 g/cm^3^	1.10–1.20 g/cm^3^
Amine number	500–700 mg KOH/g	min. 1100 mg KOH/g	290–360 mg KOH/g
Gelation time (example for composition with Epidian 5 at 20 °C, for 100 g sample)	17 min	33 min	180 min

**Table 3:** Used amount of curing agents in epoxy compositions.

Epoxy Resin	Curing Agent (Parts by Weight per 100 Parts by Weight of Epoxy Resin)		
	TFF Epoxy Resin	Z-1 Epoxy Resin	PAC Epoxy Resin
Epidian 5 (E5)	26 part-weight.	12 part-weight.	80 part-weight.
Epidian 53 (E53)	22 part-weight.	10 part-weight.	80 part-weight.
Epidian 57 (E57)	22 part-weight.	10 part-weight.	80 part-weight.

**Table 4:** Properties of NanoBent ZR2 montmorillonite [25].

Properties	ZR2 NanoBent
Form	cream-colored lamellar powder
Water content	≤3.0% weights
Roasting loss at 650 °C	25–30% weights
Swelling in Xylene	>20% volume
Vapor sorption of white spirit 48 h	>20% weights
Bulk density	<0.5 g/cm^3^
CEC ion exchange capacity of bentonite raw material	min. 80 mmol/100 g dry bentonite raw material

**Table 5:** Basic physical and chemical properties of CaCO_3_ used in the study [26].

Properties	CaCO_3_ Calcium Carbonate
Form	light gray solid of various sizes: lumps or fine powder
Fragrance	odorless
pH	9.2 (at 25 °C)
Melting point	>450 °C (decomposition temperature—825 °C)
Flammability	non-flammable
Explosive limits	non-explosive (free of any chemical structures associated with explosive properties)
Relative density	2.711 g/cm^3^ (at 20 °C)
Solubility in water	14 mg/dm^3^ (at 25 °C)
Viscosity	not applicable (solid with melting point > 450 °C)
Explosive properties	non-explosive (free of any chemical structures associated with explosive properties)
Oxidizing properties	non-oxidizing (based on chemical structure, the substance does not contain excess oxygen or any structural group tending to react exothermically with combustible material)
Decomposition temperature	825 °C
Bulk density	(0.7–1.4)·10^6^ g/m^3^ (at 20 °C)
Electrostatic properties	the substance does not generate electrostatic charges

**Table 6:** Basic properties of CWZ-22 activated carbon [27].

Properties	CWZ-22 Activated Carbon
Form	solid, dusty black color
Fragrance	odorless
pH	about 6 (50 g/L H_2_O as suspension, 20 °C)
Melting point	no data, sublimation about 3700 °C
Explosive limits	no data
Relative density	about 2 g/cm^3^
Solubility in water	in water: insoluble in organic solvents: no data
Bulk density	about 400·10^3^ g/m^3^

**Table 7:** Tested compositions based on epoxy resin Epidian 5.

No	Epoxy Resin			Curing Agent			Filler		
	Epidian 5	Epidian 53	Epidian 57	TFF	Z-1	PAC	ZR-2	CaCO_3_	CWZ-22
1	100	0	0	26	0	0	0	0	0
2	100	0	0	26	0	0	1	0	0
3	100	0	0	26	0	0	3	0	0
4	100	0	0	26	0	0	5	0	0
5	100	0	0	26	0	0	0	5	0
6	100	0	0	26	0	0	0	10	0
7	100	0	0	26	0	0	0	20	0
8	100	0	0	26	0	0	0	0	5
9	100	0	0	26	0	0	0	0	10
10	100	0	0	26	0	0	0	0	20
11	100	0	0	0	12	0	0	0	0
12	100	0	0	0	12	0	1	0	0
13	100	0	0	0	12	0	3	0	0
14	100	0	0	0	12	0	5	0	0
15	100	0	0	0	12	0	0	5	0
16	100	0	0	0	12	0	0	10	0
17	100	0	0	0	12	0	0	20	0
18	100	0	0	0	12	0	0	0	5
19	100	0	0	0	12	0	0	0	10
20	100	0	0	0	12	0	0	0	20
21	100	0	0	0	0	80	0	0	0
22	100	0	0	0	0	80	1	0	0
23	100	0	0	0	0	80	3	0	0
24	100	0	0	0	0	80	5	0	0
25	100	0	0	0	0	80	0	5	0
26	100	0	0	0	0	80	0	10	0
27	100	0	0	0	0	80	0	20	0
28	100	0	0	0	0	80	0	0	5
29	100	0	0	0	0	80	0	0	10
30	100	0	0	0	0	80	0	0	20

**Table 8:** Tested compositions based on epoxy resin Epidian 53.

No	Epoxy Resin			Curing Agent			Filler		
	Epidian 5	Epidian 53	Epidian 57	TFF	Z-1	PAC	ZR-2	CaCO_3_	CWZ-22
1	0	100	0	22	0	0	0	0	0
2	0	100	0	22	0	0	1	0	0
3	0	100	0	22	0	0	3	0	0
4	0	100	0	22	0	0	5	0	0
5	0	100	0	22	0	0	0	5	0
6	0	100	0	22	0	0	0	10	0
7	0	100	0	22	0	0	0	20	0
8	0	100	0	22	0	0	0	0	5
9	0	100	0	22	0	0	0	0	10
10	0	100	0	22	0	0	0	0	20
11	0	100	0	0	10	0	0	0	0
12	0	100	0	0	10	0	1	0	0
13	0	100	0	0	10	0	3	0	0
14	0	100	0	0	10	0	5	0	0
15	0	100	0	0	10	0	0	5	0
16	0	100	0	0	10	0	0	10	0
17	0	100	0	0	10	0	0	20	0
18	0	100	0	0	10	0	0	0	5
19	0	100	0	0	10	0	0	0	10
20	0	100	0	0	10	0	0	0	20
21	0	100	0	0	0	80	0	0	0
22	0	100	0	0	0	80	1	0	0
23	0	100	0	0	0	80	3	0	0
24	0	100	0	0	0	80	5	0	0
25	0	100	0	0	0	80	0	5	0
26	0	100	0	0	0	80	0	10	0
27	0	100	0	0	0	80	0	20	0
28	0	100	0	0	0	80	0	0	5
29	0	100	0	0	0	80	0	0	10
30	0	100	0	0	0	80	0	0	20

**Table 9:** Tested compositions based on epoxy resin Epidian 57.

No	Epoxy Resin			Curing Agent			Filler		
	Epidian 5	Epidian 53	Epidian 57	TFF	Z-1	PAC	ZR-2	CaCO_3_	CWZ-22
1	0	0	100	22	0	0	0	0	0
2	0	0	100	22	0	0	1	0	0
3	0	0	100	22	0	0	3	0	0
4	0	0	100	22	0	0	5	0	0
5	0	0	100	22	0	0	0	5	0
6	0	0	100	22	0	0	0	10	0
7	0	0	100	22	0	0	0	20	0
8	0	0	100	22	0	0	0	0	5
9	0	0	100	22	0	0	0	0	10
10	0	0	100	22	0	0	0	0	20
11	0	0	100	0	10	0	0	0	0
12	0	0	100	0	10	0	1	0	0
13	0	0	100	0	10	0	3	0	0
14	0	0	100	0	10	0	5	0	0
15	0	0	100	0	10	0	0	5	0
16	0	0	100	0	10	0	0	10	0
17	0	0	100	0	10	0	0	20	0
18	0	0	100	0	10	0	0	0	5
19	0	0	100	0	10	0	0	0	10
20	0	0	100	0	10	0	0	0	20
21	0	0	100	0	0	80	0	0	0
22	0	0	100	0	0	80	1	0	0
23	0	0	100	0	0	80	3	0	0
24	0	0	100	0	0	80	5	0	0
25	0	0	100	0	0	80	0	5	0
26	0	0	100	0	0	80	0	10	0
27	0	0	100	0	0	80	0	20	0
28	0	0	100	0	0	80	0	0	5
29	0	0	100	0	0	80	0	0	10
30	0	0	100	0	0	80	0	0	20

**Table 10:** Hyperparameters and structural details of the machine learning models.

Model Hyperparameters	Neural Network Model for Tensile Strength [MPa]	SVM Model for Compression Strength [MPa]	Neural Network Model Bending Strength [MPa]
Prediction speed (obs/s)	73,000	23,000	67,000
Training time (s)	2.5936	2.6161	1.51
Layers	2	N/A	3
Layer sizes	10-10	N/A	10-10-10
Activation	ReLU	N/A	ReLU
Regularization	λ = 0	kernel scale (RBF kernel) = 0.75 (γ ≈ 0.889); box constraint C ≈ 100 (auto); epsilon ≈ 2.5 MPa (auto)	λ = 0
Standardize data	Yes	Yes	Yes

**Table 11:** Model performance metrics.

Model Results	Neural Network Model for Tensile Strength [MPa]	SVM Model for Compression Strength [MPa]	Neural Network Model for Bending Strength [MPa]
R-Squared (Validation)	0.64	0.93	0.95
RMSE (Validation)	4483.5	6.4227	6.6236
MSE (Validation)	20,102,000.0	41.251	43.872
MAE (Validation)	694.66	3.8083	4.317
MAPE (Validation)	1494.2%	5.0%	7.1%

## Data Availability

The original contributions presented in this study are included in the article. Further inquiries can be directed to the authors.
